# Contrasting Effects of Different Mammalian Herbivores on Sagebrush Plant Communities

**DOI:** 10.1371/journal.pone.0118016

**Published:** 2015-02-11

**Authors:** Kari E. Veblen, Kyle C. Nehring, Christopher M. McGlone, Mark E. Ritchie

**Affiliations:** 1 Ecology Center and Department of Wildland Resources, Utah State University, Logan, Utah, United States of America; 2 USDA-ARS Pollinating Insect Research Unit, Utah State University, Logan, Utah, United States of America; 3 Department of Biology, Syracuse University, Syracuse, New York, United States of America; Helmholtz Centre for Environmental Research (UFZ), GERMANY

## Abstract

Herbivory by both grazing and browsing ungulates shapes the structure and functioning of terrestrial ecosystems worldwide, and both types of herbivory have been implicated in major ecosystem state changes. Despite the ecological consequences of differences in diets and feeding habits among herbivores, studies that experimentally distinguish effects of grazing from spatially co-occurring, but temporally segregated browsing are extremely rare. Here we use a set of long-term exclosures in northern Utah, USA, to determine how domestic grazers vs. wild ungulate herbivores (including browsers and mixed feeders) affect sagebrush-dominated plant communities that historically covered ~62 million ha in North America. We sampled plant community properties and found that after 22 years grazing and browsing elicited perceptible changes in overall plant community composition and distinct responses by individual plant species. In the woody layer of the plant community, release from winter and spring wild ungulate herbivory increased densities of larger Wyoming big sagebrush (*Artemisia tridentata*, ssp. *wyomingensis*) at the expense of small sagebrush, while disturbance associated with either cattle or wild ungulate activity alone was sufficient to increase bare ground and reduce cover of biological soil crusts. The perennial bunchgrass, bottlebrush squirretail (*Elymus elymoides*), responded positively to release from summer cattle grazing, and in turn appeared to competitively suppress another more grazing tolerant perennial grass, Sandberg’s blue grass (*Poa secunda*). Grazing by domestic cattle also was associated with increased non-native species biomass. Together, these results illustrate that ungulate herbivory has not caused sagebrush plant communities to undergo dramatic state shifts; however clear, herbivore-driven shifts are evident. In a dry, perennial-dominated system where plant community changes can occur very slowly, our results provide insights into potential long-term trajectories of these plant communities under different large herbivore regimes. Our results can be used to guide long-term management strategies for sagebrush systems and improve habitat for endemic wildlife species such as sage-grouse (*Centrocercus* spp.).

## Introduction

Large ungulate herbivory shapes the physiognomy and functioning of terrestrial ecosystems worldwide. Both grazing and browsing can profoundly influence the structure and composition of plant communities [1–6] with far-reaching consequences for multiple taxa [7–11], including other ungulate herbivores [12–15]. The presence of grazers, such as cattle, has been implicated in major ecosystem state shifts, including woody plant encroachment and apparently permanent conversions from native to non-native plant communities [16–19] (but see [20–22]). Yet despite the potential for additive or antagonistic effects among different ungulates on plant communities (e.g., [23]), the effects of browsing have been underemphasized in the scientific grazing literature and often times completely ignored.

**Fig 1:**
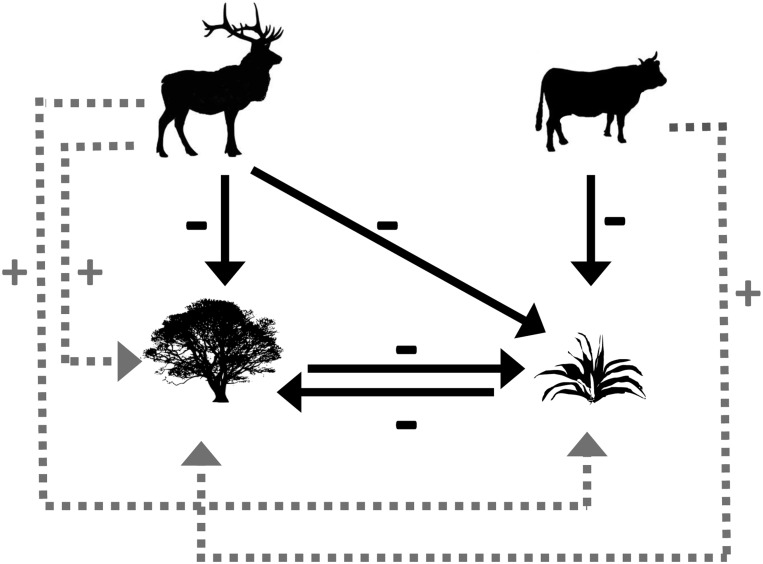
Conceptual model. Conceptual model of interactions among browsing, grazing, woody plants, and perennial grasses. Model assumes that wild ungulates (top left) will both browse and graze, while cattle (top right) will primarily graze. Solid arrows indicate direct effects and assume net negative effects of herbivory and competition on plant biomass. Dashed arrows indicate indirect effects of browsing and grazing mediated through competition between woody plants (bottom left) and perennial grasses (bottom right). Browsing and grazing, depending on their timing, intensities, interactions, and net effects, can shift plant communities between woody- and grass-dominated states.

Large herbivores can have contrasting effects on plant community composition and dynamics through their behavior and diet preferences [24, 25], both of which can vary seasonally [26, 27]. Cattle are globally distributed domestic grazers that efficiently digest herbaceous material (i.e., mostly grasses and some forbs). Heavy use by grazers such as cattle can reduce or eliminate perennial grasses, thereby shifting plants towards dominance by woody species (Fig. 1) [28–30] or less palatable herbaceous species [31, 32]. While some wild ungulate grazers (e.g., equids and Bovini) may have effects on plant communities very similar to domestic cattle, other wild ungulates are browsers (e.g., North American mule deer [*Odocoileus hemionus*]) that feed primarily on woody species and forbs [33] or mixed feeders (e.g., elk [*Cervus elaphus*]) that both graze and browse and show a variable and broad diet range [34–36]. In highly seasonal systems the feeding behavior of ungulate herbivores varies temporally whereby animals browse woody plants more in winter or spring when herbaceous forage is unavailable [27, 37]. Browsing can exert major controls over woody plant dynamics and may directly oppose the effects of grazing on state changes between woody- and grass-dominated states (Fig. 1) [38–40]. Large ungulate herbivores also have the potential to reduce plant community resistance to disturbance and invasion by undesirable plant species, for instance by preferentially foraging on the most palatable plants, increasing bare ground or damaging biological soil crusts via hoof action [41–44]. The relative roles of different types of large herbivores in invasion dynamics, however, have received little attention in the scientific literature [43].

Rarely are the effects of grazing and browsing herbivores distinguished within a single study [45–50] (but see [51]). An abundance of studies focus on the experimental effects of livestock grazing (e.g., [2, 20, 52–54]). Of those that use controlled replication, most make comparisons between treatments to which livestock grazers do vs. do not have access. Typically wild ungulates are allowed access to both treatment types, thereby controlling for, but not exploring the potentially important role of wild ungulates (which can include grazers, browsers, and/or mixed feeders) in plant community dynamics. Other studies experimentally test the effects of wild ungulates in the absence of domestic grazers [55–59] (but see [15, 54]), a land management scenario which is becoming increasingly uncommon. Experimentally investigating the roles of large herbivores with contrasting feeding habits within a single system in a controlled setting will provide insights into the mechanisms behind plant-herbivore dynamics and management options for multi-use landscapes.

Here we use a set of long-term exclosures to determine how cattle (which in our study are grazers that use the site primarily in summer) vs. wild ungulates (which in our study are browsers and mixed feeders that use the study site mainly in winter and spring) affect sagebrush-dominated plant communities. These plant communities provide critical ecological services and habitat for endemic plant and wildlife species, but presently cover less than half of their original 60 million ha in the Intermountain West of North America [60–63]. We focus on communities dominated by Wyoming big sagebrush (*Artemisia tridentata ssp. wyomingensis*), for which there are no published studies that use controlled experimentation to distinguish domestic grazer and wild ungulate (primarily Rocky mountain elk and mule deer) effects on plant community dynamics. In particular we test how different types of herbivory influence a) shrubs and grasses, the dominant components of the plant community (Fig. 1), and b) non-native species and ground cover (including biological soil crusts) that can further influence plant community dynamics.

## Materials and Methods

### Site Description

This study was conducted at Deseret Land and Livestock (DLL), a private ranch headquartered 13 km south of Woodruff, UT, USA (41° 24’ N; 111° 13’ W). Our experimental sites are dominated by Wyoming big sagebrush (*Artemisia tridentata* ssp. *wyomingensis*) and were intentionally located in lower elevation areas that are used by cattle in the spring and summer (May—September) and by wild ungulates predominantly in the winter and early spring (November—March). The primary native ungulates on DLL are elk (*Cervus elaphus*), mule deer (*Odocoileus hemionus*), and to a lesser extent, pronghorn (*Antilocarpa americana*). Both elk and deer migrate to higher elevations during the summer and descend to lower elevations in the winter. Elk are considered mixed feeders, and the proportion of their diets made up of browse increases in the winter, particularly under snowier conditions [27]. Deer, considered browsers, rely heavily on woody material in the winter [27]. The main grass species at the study site are bluebunch wheatgrass (*Pseudoroegneria spicata*), Indian ricegrass (*Achnatherum hymenoides*), Sandberg’s bluegrass (*Poa secunda*), needle and thread (*Hesperostipa comata*) and bottlebrush squirreltail (*Elymus elymoides*). The main forbs are spiny phlox (*Phlox hoodii*), pussytoes (*Antennaria* spp.), and milk vetch (*Astragalus* spp.). The primary non-natives are bur buttercup (*Ceratocephala testiculata*), desert alyssum *(Alyssum desertorum*), and cheatgrass (*Bromus tectorum*). Plant species nomenclature and nativity are based on the USDA PLANTS database [64].

Our study focused on three livestock pastures (Dip, Neponset, and Kate) ranging in size from 567–1200 ha. Neponset (1980 m, 6–10% slopes, sandy loam soils) and Kate (2010 m, 4–10% slopes, fine sandy loam soils) pastures are typically grazed on a 5 year rest-rotation schedule. These pastures are grazed by cattle in May or June for three years and in October or November for one year, and then are rested for the fifth year. Dip pasture (2010 m, 6–15% slopes, loam soils) typically is grazed in both the early (May) and late (September) growing season and is rarely rested. The average duration for grazing on each pasture is 10 days to three weeks, depending on forage production and herd size; average cattle herd size per pasture is 1000–1500 head of cattle (Mike Meek, DLL Ranch Manager, *Pers. Comm*.). Rainfall for the year (July—June) preceding our sample periods was 70.1% of the 20-year average for 2012 and 85.2% for 2013. Rainfall for the 4 months (March—June) preceding our sample periods was 25.7% of the 20-year average for 2012 and 37.8% for 2013.

### Study design

In 1991 and 1992, we established a set of three 90 m x 90 m plots in each of the three study pastures. Within each pasture, one 90 m x 90 m plot was assigned to each of the following treatment types: 1) Total exclosure (no large ungulates allowed), 2) Cattle exclosure (access by wild ungulates only), and 3) Control (access by wild ungulates and cattle). Total exclosures were constructed with 2.5 m high barbwire fencing (20 strand spaced 20 cm vertically) to fence out all ungulates. Cattle exclosures were constructed with low (1.5 m) barbwire fencing (three strands spaced 50 cm vertically) to allow wild ungulates to traverse the fence; additionally one entire side of each cattle exclosure was opened during the majority of the year (when livestock were not in the pasture) to facilitate wild ungulate access. Wild ungulate use of our study pastures is highest during November—March when wild ungulates focus their foraging activity in these lower elevation areas. Control plots were unfenced.

In July of 2012 and 2013, we sampled vegetation, ground cover, and ungulate use along five 50 m transects in the central 60 m x 60 m study area of each 90 m x 90 m plot. The five transects were ten meters apart, oriented perpendicular to the overall slope of the plot. Vegetation frequency data were collected in five evenly spaced 1 m x 1 m quadrats per transect (n = 25 per plot). We recorded densities of live and dead shrubs by height class (<15 cm, 15–50 cm, 50–100 cm, 100–200 cm) in 4 m wide belts along each transect. Densities of perennial bunchgrasses (identified to species) and cattle, elk, deer, pronghorn and lagomorph pellet groups (identified to species by shape and size) were recorded along 2 m wide belt transects. To assess water runoff and erosion potential we measured gap size between the basal growth of perennial plants along a 50 m transect (sensu [65]). We sampled ground cover (below any vegetation) in twenty-pin 25 cm^2^ pin frames. Pin frames were placed every 5 m along each transect (n = 50 per plot). We classified pin hits into five class types: biological soil crust (non-moss), biological soil crust (moss), litter, bare ground and physical (non-biological) crust.

Plant biomass data were collected from late June to mid-July in 1992 (baseline data) and again in 2012 and 2013 in the core 60 m x 60 m study areas of each plot. This core study area was subdivided into nine 20 m x 20 m subplots. In each sampling year, one of the nine subplots per exclosure was randomly selected for biomass harvesting. Within the selected subplots, all live aboveground biomass of rooted and overhanging plants was harvested per species within three randomly located 10 cm x 5 m biomass transects. The harvested biomass was sorted to only include the current year’s growth which was then dried for 48 hours at 50°C and weighed.

### Statistical Analyses

Plant community level analyses were based on frequency data which were summarized by first determining a frequency value for each transect (occurrence in 0–5 quadrats) and then averaging the five transect values to determine a plot level value. Species that occurred in <5% of the 225 plots were removed from the analyses. We used nonmetric multidimensional scaling (NMDS) to ordinate average frequency values for plant species in our 9 plots (3 treatments x 3 pastures) (S1 File). We then performed individual NMDS analyses for each pasture (3 treatments x 5 transects, in this case averaging by transect rather than plot) (S2 File). NMDS is a robust unconstrained ordination method that uses plant occurrence data alone to identify ordination axes and explain plant community variation [66]. We used the metaMDS function in the Vegan library (version 2.0–8) for R (version 3.0.1). This function produces ordinations based on multiple random starts to avoid local minima and rotates axes to maximize variance along the first axis. We also used the Adonis function to perform a permutational multivariate analysis of variance (permANOVA) testing simultaneous response of all plant species to a) the effects of Pasture and Treatment and b) the effects of Treatment within each Pasture (permutations = 999). We set alpha values of 0.1 and used Bray-Curtis (Sorenson) dissimilarity matrices for all analyses.

Plant biomass data were organized into five growth forms: Shrubs, Grasses, Forbs, Non-native annuals, and Total (i.e., all growth forms combined). These data were analyzed with generalized linear mixed models (GLMMs) [67]. Fixed effects included herbivore treatment (Total exclosure, Cattle exclosure, or Control), year (2012 or 2013), treatment*year interaction, baseline biomass (measured in 1992), and treatment*baseline biomass interaction. Non-significant (p > 0.1) treatment*baseline biomass interactions were removed from models. Random factors included pasture and plot nested within pasture, and we used an autoregressive AR(1) covariance structure to address the non-independence of repeated surveys within the same subplot. Response variables were variance-weighted when necessary to meet model assumptions, and Tukey post-hoc tests were used. Analyses were run in R 3.0.1 (package nlme [67]). Results are reported as untransformed means ± 1 S.E. We then performed Cohen’s *d* effect size analyses (described below) for each growth form in each year.

We used Cohen’s *d* (also known as Hedges’ *g*) effect size analysis [68, 69] for analysis of all other data. A *d* statistic was calculated separately for Cattle vs. Control and Total vs. Control comparisons within each pasture. We calculated *d* statistics using the following equation:

$$d\;=\frac{m_{2}-\;m_{1}}{s_{\text{pooled}},}$$

where the pooled standard deviation (*s*_pooled_) is calculated as:

$$s_{\text{pooled}}=\sqrt{\frac{({\text{n}}_{2}-\;1){\text{s}}_{2}^{2}+\;({\text{n}}_{1}-\;1){\text{s}}_{1}^{2}}{{\text{n}}_{1}+{\text{ n}}_{2}-\;2,}}$$

where m_i_ is the transect mean, n_i_ is the number of transects, $s_{i}^{2}$ is the plot variance; subscripts 1 and 2, respectively, are values from the Control plot and treatment exclosure plot (in this case, either Cattle or Total) [68, 69]. We then calculated a mean *d* statistic across pastures for 1) Cattle vs. Control and 2) Total vs. Control comparisons, and calculated a 90% confidence interval (CI) for each mean [70]. Effect size analyses are widely used to combine results from multiple studies, which is conceptually similar to combining results from multiple sites or blocks as we have done here [71].

### Ethics statement

This study took place on privately owned land. The owner, Deseret Land and Livestock, approved all field activities. No protected species were sampled.

## Results

Pellet counts (Table 1) and effect sizes analyses of pellet densities indicate that Total exclosure treatments were effective at excluding deer (CI_90_ = -14.7 to -0.5.16), elk (CI_90_ = -46.72 to -10.17), pronghorn (CI_90_ = -8.59 to -1.81), and cattle (CI_90_ = -8.3 to -5.65). Cattle exclosure treatments successfully excluded cattle (CI_90_ = -6.58 to -5.08), but allowed pronghorn (CI_90_ = -2.66 to 0.51); deer and elk use of Cattle exclosure plots were not as high as in Control plots but were still significantly greater than in Total exclosures (deer CI_90_ = -2.43 to -0.04; elk CI_90_ = -8.26 to -1.96). Lagomorphs were not excluded from Total (CI_90_ = -2.90 to 3.25) or Cattle (CI_90_ = 0.26 to 2.65) exclosure treatments. Overall use of the site by pronghorn was low relative to deer and elk (Table 1).

**Table 1:** Dung pellet groups.

	TOTAL	CATTLE	CONTROL
Deer	1.33 ± 1.33	116 ± 36.3	142.67 ± 47.68
Elk	0 ± 0	137.33 ± 9.61	265.33 ± 51.24
Pronghorn	0 ± 0	32 ± 12.86	46.67 ± 25.96
Cattle	0 ± 0	18.67 ± 5.33	130.67 ± 6.67
Lagomorphs	1120 ± 269.59	1188 ± 124.00	1086.67 ± 124.71

Mean densities (# per ha) ±1 SE for pellet groups by species across three herbivore treatments: 1) Total exclosure plots that exclude both cattle and wild ungulates, 2) Cattle exclosure plots that exclude only cattle, and 3) Control plots where no large herbivores are excluded.

### Community level analyses

**Fig 2:**
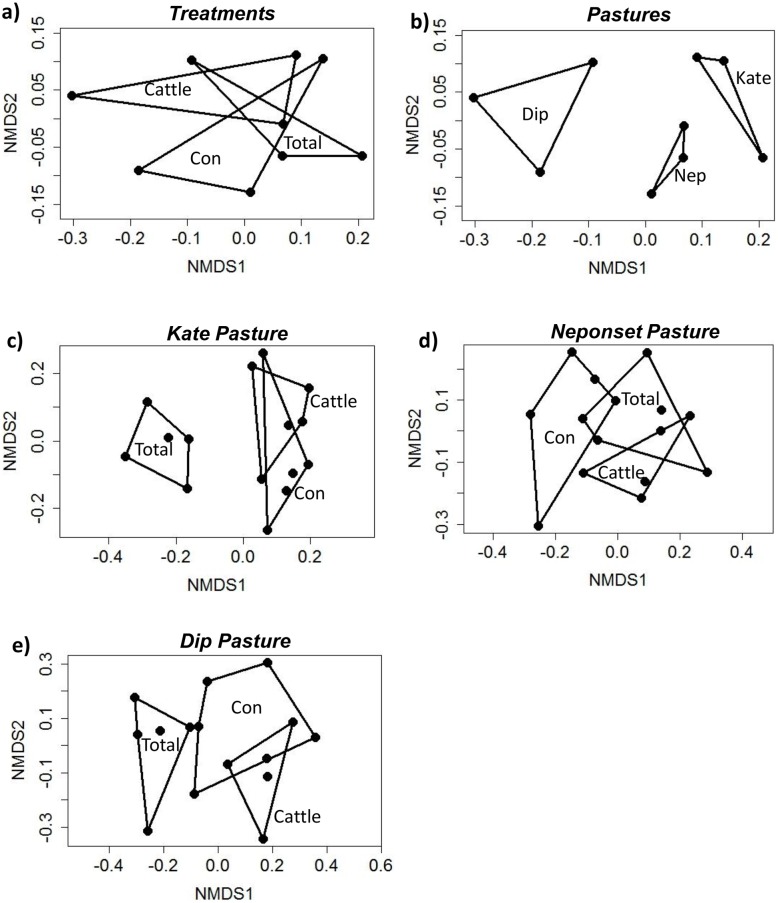
NMDS results. Non-metric Multidimensional Scaling (NMDS) results depicting sample points and convex hulls for (a) three herbivore treatments and (b) three pastures in relation to plant species community composition (n = 125 frequency frames per sample point); and (c-e) treatments within individual pastures (n = 25 frequency frames per sample point). Total, Cattle, and Control treatments exclude, respectively, all large herbivores, cattle only, and no large herbivores.

Plant community composition varied more among pastures than herbivore treatments, as indicated by a two-dimensional NMDS solution of plant frequency data that produced a good fit after one iteration (Fig. 2; stress = 0.08; R^2^ = 0.97). PermANOVA results further support these results, indicating a significant effect of “pasture” (pseudo-F_2,4_ = 0.01; Fig. 2b) and non-significant “herbivore treatment” effect (pseudo-F_2,4_ = 0.65; Fig. 2a).

NMDS plant community analyses for individual pastures, however, revealed distinctions among different herbivore treatments. Three-dimensional NMDS solutions for Kate and Dip pastures indicated that Total exclosure plots were distinct from Cattle and Control plots (Fig. 2c,e); distinctions were less clear for Neponset (Fig. 2d). Fits were good to fair (Kate stress = 0.11, R^2^ = 0.99; Neponset stress = 0.096, R^2^ = 0.91; Dip stress = 0.098; R^2^ = 0.91). Results of permANOVA indicated highly significant treatment effects in Kate (pseudo-F_2,12_ = 0.001) and Dip (pseudo-F_2,12_ = 0.001) pastures (Fig. 2c,e), and less significant treatment effects in Neponset (pseudo-F_2,12_ = 0.07) pasture (Fig. 2d).

Examination of species richness data (derived from frequency plots) across all pastures indicate that cattle reduced plant species richness. Cattle exclosure treatments reduced richness relative to Control plots (CI_90_ = -1.33 to-0.61), whereas Total exclosure treatments did not significantly alter species richness relative to Control plots (CI_90_ = -3.13 to 1.59).

### Biomass

**Fig 3:**
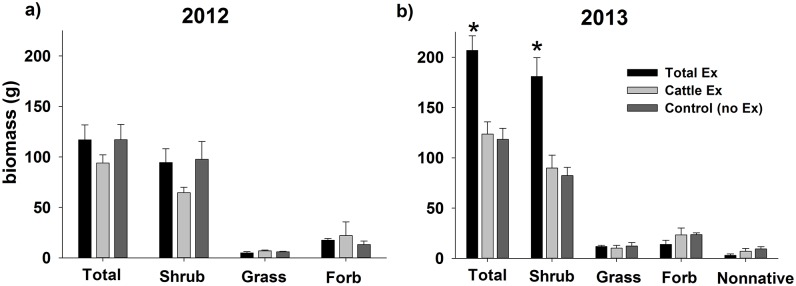
Plant Biomass. Mean biomass ± 1 S.E. in (a) 2012 and (b) 2013 of different plant growth forms (Total [i.e., all groups combined], Shrubs, Grasses, Forbs, and Non-natives) across three herbivore treatments. “Total Ex” denotes exclusion of large herbivore wildlife and cattle, “Cattle Ex” denotes exclusion of only cattle (but not wild herbivores), and “Control” denotes no exclusion i.e., access by both types of herbivore. Asterisks indicate treatment-year combinations that are significantly different (p < 0.10) than all other treatment-year combinations within a given plant growth form.

Shrub, Grass, Forb and Total biomass were significantly lower in the drier year, 2012 (Fig. 3; Shrubs F_1,6_ = 136.91, p<.0001; Grass F_1,6_ = 24.81, p = 0.003; Forbs F_1,6_ = 3.80, p = 0.099; Total biomass F_1,6_ = 17.34, p = 0.006), and Non-native biomass was too low for analysis during this year. Effect size analyses also revealed significant effects of Cattle, but not Total exclusion treatments in 2012 (Table 2). In particular, Cattle exclusion significantly increased Grass biomass relative to Controls. Cattle exclusion also decreased Total biomass, a pattern most likely driven by non-significant treatment effects on Shrub biomass (Table 2), which made up 78% of Total biomass.

**Table 2:** Biomass effect sizes.

	YEAR	2012	2013
TOTAL BIOMASS	Total	-0.32 ± 1.56	3.81 ± 3.64
	Cattle	-0.68 ± 0.56	0.59 ± 1.83
SHRUBS	Total	-0.53 ± 1.84	4.23 ± 4.48
	Cattle	-1.16 ± 1.24	0.06 ± 1.49
GRASSES	Total	-0.18 ± 1.80	-0.01 ± 0.90
	Cattle	1.51 ± 1.48	-0.007 ± 0.1.91
FORBS	Total	0.42 ± 0.58	-1.52 ± 1.04
	Cattle	-0.31 ± 2.39	-0.50 ± 1.02
NON-NATIVES	Total	—	-2.54 ± 2.41
	Cattle	—	-1.21 ± 0.91

Means and 90% confidence intervals for Cohen’s *d* effect size analyses of annual biomass in 2012 and 2013. “Total” denotes effect of exclusion of all large herbivores relative to Control plots (where no large herbivores are excluded), and “Cattle” denotes effects of exclusion of only cattle (but not wild herbivores) relative to Control plots. Bold values indicate treatments with confidence intervals that do not overlap zero and are considered significantly different from control.

The wetter year (2013) revealed significant effects of Total exclusion that were not evident in 2012. In 2013, both Total biomass and Shrub biomass were higher in Total exclosure plots than in all other treatment-year combinations (Fig. 3; Total biomass F_2,6_ = 7.27, p = 0.03; Shrubs F_2,6_ = 17.38, p = 0.003). Effect size analyses of 2013 biomass also indicate lower forb biomass in Total relative to Control plots (Table 2). The wetter year also revealed patterns in non-native species (that had been too low in biomass to analyze in the drier year). In particular, in 2013, non-native biomass (comprised of 51% *C. testiculata*, 47% *A. desertorum*, and 2% *B. tectorum*) was significantly lower in Cattle and Total exclosure plots relative to Control plots (Table 2). The overall effect size was greatest for Total treatments, but the variability was too great to be significantly different than Cattle exclosure treatments.

### Species and growth form analyses

**Fig 4:**
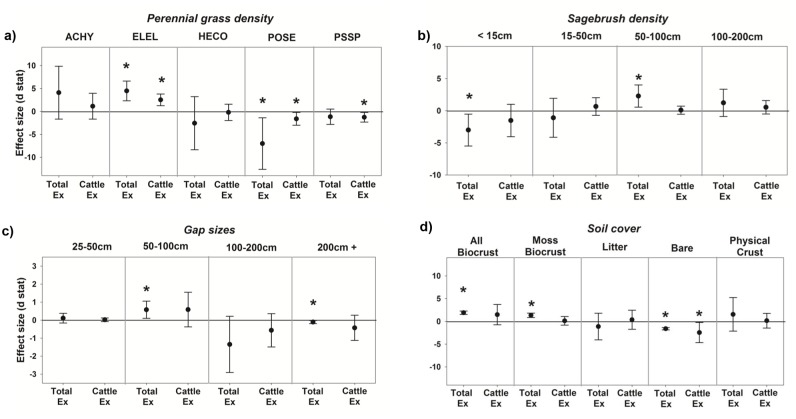
Effect size analyses. Effect size analyses of (a) perennial grass (*Achnatherum hymenoides* [ACHY], *Elymus elymoides* [ELEL], *Hesperostipa comata* [HECO], *Poa secunda* [POSE], *Pseudoroegneria spicata* [PSSP]) density, (b) sagebrush (*A. tridentata* ssp. *wyomingensis*) density, (c) inter-plant gap size, and (d) soil cover across herbivore treatments. “Total Ex” denotes exclusion of all large herbivores, and “Cattle Ex” denotes exclusion of only cattle (but not wild herbivores). If symbol is above the zero line, then magnitude of response is greater in the treatment exclosure than in the control. If symbol is below the zero line, then response is less than in the control. Error bars show 90% CI. If error bars do not equal or cross zero line, then difference is considered significant.

Densities of three perennial grasses varied with respect to herbivore treatment (Fig. 4a, Table 3). Effect size analyses indicate that densities of the perennial bunchgrass *E*. *elymoides* increased in both Cattle and Total exclosure plots compared to controls (Fig. 4a). The most common grass, *P*. *secunda*, showed the opposite pattern and was lower in the two exclosure treatments than in the controls (Fig. 4a). The grass *P*. *spicata* was reduced by cattle exclusion. *Achnatherum hymenoides* and *H*. *comata* did not respond significantly to herbivore treatment.

**Table 3:** Plant densities, gaps, and soil cover.

	TOTAL	CATTLE	CONTROL
Perennial grass density^a^			
ACHY	497.4 ± 229.6	297.4 ± 155.4	248 ± 123.6
ELEL	500 ± 69.4	364 ± 37.2	201.4 ± 60.2
HECO	553.4 ± 268.2	1036 ± 544.6	1208 ± 548
POSE	1898.6 ± 368.6	2462.6 ± 436	2758.6 ± 379.2
PSSP	259 ± 188.6	147.8 ± 61.6	301.6 ± 119.6
Sagebrush density^a^			
<15 cm	42 ± 4.2	90.7 ± 2.4	458.7 ± 366.5
15–50 cm	821.3 ± 178	1098 ± 349.9	952.7 ± 260.5
50–100 cm	1253.3 ± 142.2	993.3 ± 138.6	994.7 ± 95.5
100–200 cm	44.7 ± 31.7	54.7 ± 47.8	51.3 ± 49.3
Inter-plant gaps^b^			
25–50 cm	74.12 ± 3.88	72.7 ± 1.53	78.13 ± 1.42
50–100 cm	64.8 ± 6.31	62.32 ± 3.08	65.96 ± 3.89
100–200 cm	43.18 ± 7.53	37.58 ± 5.24	44.63 ± 5.57
200+ cm	19.6 ± 5.81	15.3 ± 4.41	18.31 ± 5.86
Soil cover^c^			
All biocrust	2.66 ± 0.25	2.16 ± 0.25	1.62 ± 0.15
Moss biocrust	1.84 ± 0.27	1.27 ± 0.13	1.27 ± 0.12
Litter	11.75 ± 0.94	13.36 ± 0.67	12.69 ± 0.94
Bare	1.44 ± 0.22	1.31 ± 0.22	2.06 ± 0.15
Physical crust	4.59 ± 1.11	3.67 ± 0.28	3.89 ± 0.98

Means ±1 SE for perennial grass densities by species, sagebrush (*Artemisia tridentata* ssp. *wyomingensis*) densities by size class, inter-plant gaps by total length in each gap size class, and soil cover types across three herbivore treatments: 1) Total exclosure plots that exclude both cattle and wild ungulates, 2) Cattle exclosure plots that exclude only cattle, and 3) Control plots where no large herbivores are excluded.

^a^ #/ha

^b^ total cm

^c^ # pin hits (of 20)

Densities of *A. tridentata* ssp. *wyomingensis* varied across herbivore treatments and shrub size classes (Fig. 4b, Table 3). The Total herbivore exclusion treatment, the only treatment to exclude wildlife, was characterized by a higher number of mid-sized (50–100 cm) individuals and lower number of small (<15 cm) individuals relative to control plots. The Cattle exclusion treatment (to which wildlife did have access), did not significantly differ from Control. There were no significant effect size differences for the 15–50 cm and 100–200 cm size classes, though they followed similar trends to <15 cm and 50–100 cm size classes, respectively.

Gap size analyses indicate that plots where both cattle and wildlife were excluded (i.e., Total exclusion plots) had a significantly higher proportion of 50–100 gaps, but lower proportion of very large (200 cm+) gaps (Fig. 4c). The infilling of very large gaps (and resulting increase in smaller gaps) is consistent with the absolute increases in densities of 50–100 cm *A. tridentata* ssp. *wyomingensis* shrubs (Table 3) and the grass *E. elymoides* (Table 3) in Total exclosure plots relative to control plots.

Effect size analyses indicated that cover of all biological soil crust groups combined was increased by Total exclusion plots, likely driven by moss, the only group with cover high enough for individual analysis (Fig. 4d). Amount of bare soil was significantly lower in both treatments that excluded cattle (Fig. 4d). There were no significant effect size differences for either litter or physical crust (Fig. 4d).

## Discussion

Grazing and browsing ungulates co-habit rangelands worldwide and can have distinct and sometimes contrasting effects on plant community trajectories [45, 48, 49, 72, 73]. This is the first published study to experimentally address and demonstrate, within a single study, the effects of long-term manipulation of both wild ungulates (including browsers and mixed feeders) and domestic grazers on Wyoming big sagebrush (*Artemisia tridentata* ssp. *wyomingensis*), a dominant land cover type of conservation concern [60–63] in the western U.S.A. Here we have revealed, after only twenty-two years, distinct effects of a domestic grazer and native ungulates on major plant species and growth forms, including long-lived native perennial species, annual invasive species, and biological soil crusts. These changes to specific plant functional groups and soil crusts were accompanied by perceptible effects of wild ungulate exclusion on overall plant community composition (as assessed by NMDS) in this sagebrush community. At two of three sites, overall plant community composition differed in the one treatment that excluded vs. two treatments that allowed wild ungulate access (Fig. 2c,e), illustrating a clear wild ungulate effect and a relatively small domestic grazer effect. Distinctions among the three herbivore treatments were weaker (but still significant to the 0.1 level) for the third site (Fig. 2d), possibly because of greater heterogeneity in soil type and landscape position among plots, or because of lower overall plant productivity (pers. obs) and herbivore use (Table 1) at the site.

### Specific wildlife effects

Wildlife effects on plant community composition appeared to be driven at least partially by browsing of woody species. Wild ungulates showed size-class-specific effects on Wyoming big sagebrush (*A. tridentata* ssp. *wyomingensis*) densities across all sites. The dominant herbivores in the study area that browse on woody species in the winter are elk and mule deer. As might be expected from examples of browsing effects in a variety of ecosystems [38–40, 74], release from browsing increased densities of the most common adult-sized sagebrush (50–100 cm) in our study. This coincided with lower relative densities of small sagebrush (<15 cm). Small sagebrush in wildlife exclusion plots likely represent mostly new recruits that were suppressed by either a) adult shrubs or b) grasses (sensu [75]) that were also released from herbivory in wildlife exclusion plots. Small sagebrush in wildlife accessible plots on the other hand likely represent a combination of new recruits and older individuals maintained in short stature by recurrent browsing [38, 76]. Regardless, our results reveal that removal of browsing animals—a phenomenon that could occur due to unintentional species losses or intentional management decisions—increases dominance by larger-statured woody species and presumably competition for resources (e.g., light) necessary for establishment and growth of smaller individuals. This pattern is typical of “bush encroachment” which is often (but not always) associated with rangeland degradation regionally and globally [19, 77, 78], and in this system could negatively affect endemic wildlife species such as sage-grouse [79].

Release from wild ungulate herbivory also increased annual biomass of shrubs (Fig. 3b), of which the majority (78–79%) were Wyoming big sagebrush. These results suggest a suppressive effect of browsing in the wildlife-accessible plots. This pattern was only evident during the wetter year (2013), perhaps because both production and browsing were lower across all treatments during the drier year. Although it is not possible to parse out the relative contributions of long-term changes in shrub density (described above) vs. short-term browsing effects on annual shrub biomass patterns, it is clear that shrub biomass is greater in the absence of browsers.

Interestingly, although woody species can suppress grasses [19, 77, 78], in this case grass production was not reduced by increased shrub production. This result provides further evidence that some perennial grasses—at least when they are ungrazed—are not necessarily out-competed by woody species [80, 81], particularly under favorable moisture conditions. Alternatively, release from grazing (by cattle, elk, or both) may have compensated for any shrub-driven reductions in grass production. Forb production, on the other hand, appeared to be reduced by shrubs, though sagebrush effects on forbs have been reported to range from neutral [80] to facilitative elsewhere [82, 83]. Forbs are especially important to management of sagebrush systems due to their potential to offer resistance against undesirable invasive annual plants such as cheatgrass (*B. tectorum*) [84] and to their role in the diets of species of concern such as sage-grouse [79]. Our results provide evidence that managing for sufficient wild ungulate populations may be a critical component of maintaining productive and structurally diverse sagebrush stands and key habitat characteristics for native species conservation.

### Specific cattle effects

Cattle grazing influenced individual species and certain plant growth forms within the herbaceous plant community. Although these effects were not yet associated with significant differences in overall plant community composition, they provide insights into potential plant community trajectories and future communities. The early successional shallow-rooted bunchgrass, bottlebrush squirretail (*E. elymoides*), showed a positive response to release from cattle grazing and in turn appeared to competitively suppress the more common shallow-rooted grass, Sandberg’s blue grass (*P. secunda*). Increased *P. secunda* dominance under grazed conditions is a regionally common pattern for this abundant, grazing-tolerant grass [85] (but see [41]). Another bunchgrass, needle and thread (*H. comata*), did not respond significantly to grazing exclusion, likely because it is only a moderately preferred forage species for livestock [64]. Although densities of the more grazing-sensitive perennial bunchgrass, bluebunch wheatgrass (*P. spicata*) would have been expected to increase under grazing release in both herbivore exclusion plot types [85], densities did not increase in Total exclusion plots, likely due to increased shrub competition. Most surprising was that densities of *P. spicata* were significantly reduced in Cattle exclusion plots relative to grazed Control plots. Historically, elk (mixed feeders) may have preferentially grazed in Cattle exclusion plots (compared to Controls) to avoid competition with cattle, thereby increasing grazing pressure on highly palatable *P. spicata*. Cattle-wildlife competition and compensatory increases in wildlife habitat use have been shown in other exclosure studies [14, 73]. In the case of our study, historic compensatory responses by elk (and the consequent reduction in desirable *P. spicata* forage) may help explain the slight present-day aversion elk showed to our cattle exclusion plots (Table 1). These types of patterns can only be revealed by study designs that explicitly distinguish effects of different types of herbivory.

Our study also revealed year-dependent effects of cattle grazing. Release from cattle grazing increased grass biomass during the drier year, 2012, when forage would have been most limiting and grazing pressure highest across the landscape. In the same plots, however, total biomass decreased (relative to Control plots), likely reflecting a (non-significant) decrease in shrub production associated with increased grass competition [86].

### Additive and synergistic cattle-wildlife effects

Due to our experimental design and because Total and Cattle exclusion effect sizes did not differ significantly from each other, we cannot rule out the possibility that the effects of “wild ungulate” removal discussed above are actually additive or synergistic effects of removing both cattle and wild ungulates from Total exclosure plots. For example, although cattle are considered “grazers” they can browse on woody material [87], and perhaps shrubs only respond positively when they are released from both wild and domestic herbivore browsing. Plant community responses in Cattle exclusion plots also may reflect more than the direct effects of removing cattle. Plant community responses in these plots may be the product of synergistic cattle-wild ungulate effects. For example, as described above for *P. spicata*, the plant community effects of cattle removal may elicit compensatory behavioral responses by wild ungulates.

Different seasonal use patterns by wild ungulates vs. cattle also can result in apparent non-additivity of herbivory effects. Whereas cattle grazing occurred during active growing periods, grazing effects of the mixed feeder at our study site, elk, would have occurred during winter and early spring when grasses and are forbs are dormant. This timing would have mitigated negative wild ungulate effects on herbaceous plants. Likewise, winter (dormant season) use of the study area by wild ungulates may have amplified negative effects of browsing on shrubs by inhibiting shrub regrowth [38] or compensatory responses like secondary chemical defenses against herbivory [88]. In addition, winter herbivory can result in net nutrient inputs to soils that stimulate grass production or quality and improve grass compensatory response to grazing later in the growing season [89]. The seasonal dichotomy in range use by wild vs. domestic livestock is widespread across western North America and in other systems dominated by *Artemisia* [90], suggesting that the overall browsing and grazing impacts we observed may be typical for sagebrush communities.

### Invasive species, inter-plant gap sizes, and biological soil crusts

One potential source of concern for land managers is that non-native species biomass was higher in plots accessible by domestic grazers during the wetter year. Exotic species invasions are arguably the primary threat to sagebrush-dominated systems in the Intermountain West of North America [91], and inappropriate livestock management practices as well as overabundance of wild herbivores are cited as contributing to invasions by undesirable species in ecosystems worldwide [5, 16, 43, 44, 92, 93]. In the present study we have not shown evidence of widespread invasion attributable to grazing; rather we have shown evidence of Wyoming big sagebrush plant communities with a small non-native species component that is more strongly expressed during wetter years—but most strongly in the presence of domestic grazers. However, even low levels of invasion can become more problematic following major disturbance (e.g., extended drought), and those species that are limited by elevation or moisture [42] may increase in prevalence in warmer climate conditions predicted for this area [94]. Moreover, cattle grazing had a negative effect on *E. elymoides*, a species that is a strong competitor with invasive *B. tectorum*. A populous and more diverse native plant community is a potential form of resistance against invasive species [95] (but see [93]) and we did find lower overall plant species richness in plots accessible by cattle.

Smaller inter-plant gap sizes between perennial plants often are associated with increased biotic resistance to disturbance and invasive species [41, 96]. We found that gap sizes were smaller in plots where wildlife were excluded, consistent with our result of higher shrub cover in these plots. Despite the potential for small inter-plant gaps to provide resistance against non-native species [41], increased invasive biomass in our study was associated with cattle rather than wild ungulate activity. This suggests that, when wild herbivores are present, and/or when the plant community is relatively uninvaded, gap size may not be an ideal early indicator or correlate of invasion risk.

Cover of biological soil crusts, an indicator of soil condition [97, 98], was higher in the Total herbivore exclusion treatment. Crusts create microsites for germination of a diversity of native plants that are typically desired for their conservation value and resistance against exotic species [41, 99, 100]. Our results suggest that the presence of wild ungulates alone (i.e., even in the absence of cattle) creates sufficient disturbance to limit biological soil crust cover. Given that the site likely evolved under wildlife herbivory (including grazers, browsers and mixed feeders), however, the level of soil crust disturbance we detected likely falls within the natural range of variability for the area. Cattle exclusion plots, on the other hand, did not significantly increase soil crust cover (relative to Control plots). But we cannot rule out cattle activity as an important driver of biological soil crust cover because cattle accessible plots showed significantly higher bare ground, which would have been driven at least in part by non-significant loss of soil crust cover. Cattle activity in other ecosystems has been shown to decrease biological soil crust cover and increase safe sites for establishment of one pervasive invader, *B. tectorum* [41, 85, 101], though it is possible that our study area is more resilient to crust disturbance because it is wetter and more productive. Nonetheless, our results suggest an important consideration in managing for biological soil crusts is total large herbivore pressure, particularly livestock which are more readily managed than wildlife. Continued efforts to untangle the effects of large ungulates on biological soil crusts are important throughout sagebrush-dominated systems that typically support both free-roaming wild ungulate herbivores and domestic livestock.

## Conclusions

Although twenty-two years of herbivore treatments did not cause dramatic state shifts in this sagebrush community, they did reveal distinctive domestic grazer vs. wild ungulate effects on plant communities and insights into potential future trajectories of these plant and soil crust communities. Our results point to the importance of wild ungulate suppression of woody plant densities and sizes. Combined with the positive wildlife effects on forbs, these results suggest that long-term conservation of Wyoming big sagebrush plant communities may require sufficient wildlife browsing to maintain productive sagebrush stands. Also of note are the positive effects of cattle activity on non-native biomass and negative effects on the native bunchgrass, *E. elymoides*. This native species and its congeners are relatively good competitors against the widespread invasive annual *B. tectorum* in sagebrush systems [102, 103].

Our results also provide evidence that herbivore effects on plant communities, including invasive species, can vary considerably across superficially similar site conditions in Wyoming big sagebrush plant communities [1, 20, 104]. Our experimental approach allowed us to identify a role of wild ungulates (which often is conflated with cattle) in shrub-grass dynamics. Valuable future work would entail using a similar approach to examine sagebrush sites across a broad range of environmental conditions and identify mechanisms for cross-site variability of herbivory effects.

## Supporting Information

S1 File Plant frequency by treatment. Mean plant frequencies for each of three treatments (DX = no large ungulates allowed, LX = cattle excluded, accessible by wild ungulates only, OX = accessible by cattle and wild ungulates) for each of three sites (Kate, Dip, Neponset). Each treatment plot had five transects with five 1 m x 1 m quadrats per transect. Species codes follow USDA Plant Database codes (http://plants.usda.gov/); AF53 is an unidentified annual forb.
(DOCX)

S2 File Plant frequency by transect. Plant frequencies for five transects (n = five 1 m x 1 m quadrats per transect) at each of three treatments (DX = no large ungulates allowed, LX = cattle excluded, accessible by wild ungulates only, OX = accessible by cattle and wild ungulates) for each of three sites (Kate, Dip, Neponset). Species codes follow USDA Plant Database codes (http://plants.usda.gov/); AF53 is an unidentified annual forb.
(DOCX)

## References

[1] Bakker ES, Ritchie ME, Olff H, Milchunas DG, Knops JMH (2006) Herbivore impact on grassland plant diversity depends on habitat productivity and herbivore size. Ecol. Lett. 9: 780–788. 16796567 10.1111/j.1461-0248.2006.00925.x

[2] Cipriotti PA, Aguiar MR (2012) Direct and indirect effects of grazing constrain shrub encroachment in semi-arid Patagonian steppes. App. Veg. Sci. 15: 35–47.

[3] Hobbs NT (1996) Modification of ecosystems by ungulates. J. Wildl. Manage. 60: 695–713.

[4] Stewart KM, Bowyer RT, Kie JG, Dick BL, Ruess RW (2009) Population density of North American elk: effects on plant diversity. Oecologia 161: 303–312. 10.1007/s00442-009-1376-z 19484268

[5] Milchunas DG, Lauenroth WK (1993) Quantitative Effects of Grazing on Vegetation and Soils over a Global Range of Environments. Ecol. Monogr. 63: 327–366.

[6] Cote SD, Rooney TP, Tremblay JP, Dussault C, Waller DM (2004) Ecological impacts of deer overabundance. Annual Review of Ecology Evolution and Systematics 35: 113–147.

[7] Keesing F, Young TP (2014) Cascading Consequences of the Loss of Large Mammals in an African Savanna. Bioscience 64: 487–495.

[8] Newbold TAS, MacMahon JA (2008) Consequences of cattle introduction in a shrubsteppe ecosystem: indirect effects on desert horned lizards (*Phyrnosoma platyrhinos*) *West* . N. Am. Nat. 68: 291–302.

[9] Beever EA, Brussard PF (2000) Examining ecological consequences of feral horse grazing using exclosures. West. N. Am. Nat. 60: 236–254.

[10] Wilkerson ML, Roche LM, Young TP (2013) Indirect effects of domestic and wild herbivores on butterflies in an African savanna. Ecol. Evol. 3: 3672–3682. 10.1002/ece3.744 24198932 PMC3810867

[11] Parsons EWR, Maron JL, Martin TE (2013) Elk herbivory alters small mammal assemblages in high-elevation drainages. J. Anim. Ecol. 82: 459–467. 23163813 10.1111/1365-2656.12009

[12] Loeser MR, Mezulis SD, Sisk TD, Theimer TC (2005) Vegetation cover and forb responses to cattle exclusion: Implications for pronghorn. Rangeland Ecol. Manage. 58: 234–238.

[13] Wagoner SJ, Shipley LA, Cook RC, Hardesty L (2013) Spring cattle grazing and mule deer nutrition in a bluebunch wheatgrass community. J. Wildl. Manage. 77: 897–907.

[14] Odadi WO, Karachi MK, Abdulrazak SA, Young TP (2011) African wild ungulates compete with or facilitate cattle depending on season. Science 333: 1753–1755. 10.1126/science.1208468 21940896

[15] Hobbs NT, Baker DL, Bear GD, Bowden DC (1996) Ungulate grazing in sagebrush grassland: Mechanisms of resource competition. Ecol. Appl. 6: 200–217.

[16] DiTomaso JM (2000) Invasive weeds in rangelands: Species, impacts, and management. Weed Sci. 48: 255–265.

[17] Mack RN (1981) Invasion of *Bromus tectorum* L into western North-America—an ecological chronicle. Agro-Ecosystems 7: 145–165.

[18] Knapp PA (1996) Cheatgrass (*Bromus tectorum* L) dominance in the Great Basin Desert—History, persistence, and influences to human activities. Global Environ. *Change* 6: 37–52.

[19] Archer S (2010) Rangeland conservation and shrub encroachment: new perspectives on an old problem In: du Toit J. T., Kock R. and Deutsch J., editors. Wild rangelands: conserving wildlife while maintaining livestock in semi-arid ecosystems. Oxford: Wiley-Blackwell pp. 53–97.

[20] Stohlgren TJ, Schell LD, Vanden Heuvel B (1999) How grazing and soil quality affect native and exotic plant diversity in rocky mountain grasslands. Ecol. Appl. 9: 45–64.

[21] Adler PB, Milchunas DG, Sala OE, Burke IC, Lauenroth WK (2005) Plant traits and ecosystem grazing effects: Comparison of US sagebrush steppe and Patagonian steppe. Ecol. Appl. 15: 774–792.

[22] Watson IW, Thomas PWE, Fletcher WJ (2007) The first assessment, using a rangeland monitoring system, of change in shrub and tree populations across the arid shrublands of Western Australia. Rangeland J. 29: 25–37.

[23] Goheen JR, Palmer TM, Keesing F, Riginos C, Young TP (2010) Large herbivores facilitate savanna tree establishment via diverse and indirect pathways. J. Anim. Ecol. 79: 372–382. 10.1111/j.1365-2656.2009.01644.x 20039982

[24] Veblen KE, Young TP (2010) Contrasting effects of cattle and wildlife on the vegetation development of a savanna landscape mosaic. J. Ecol. 98: 993–1001.

[25] Callaway RM, Walker LR (1997) Competition and facilitation: a synthetic approach to interactions in plant communities. Ecology 78: 1958–1965.

[26] Odadi WO, Karachi MK, Abdulrazak SA, Young TP (2013) Protein supplementation reduces non-grass foraging by a primary grazer. Ecol. Appl. 23: 455–463. 23634594 10.1890/12-0878.1

[27] Christianson DA, Creel S (2007) A review of environmental factors affecting elk winter diets. J. Wildl. Manage. 71: 164–176.

[28] Reiner RJ, Urness PJ (1982) Effect of grazing horses managed as manipulators of big game winter range. J. Range Manage. 35: 567–571.

[29] Briske DD, Richards JH (1995) Plant responses to defoliation: a physiological, morphological, and demographic evaluation In: Bedunah D. J. and Sosebee R. E., editors. Wildland plants: physiological ecology and developmental morphology. Denver, CO: Society for Range Management pp. 635–710.

[30] Moe S, Rutina L, Hytteborn H, du Toit J (2014) Impala as controllers of elephant-driven change within a savanna ecosystem In: Skarpe C., du Toit J. and Moe S., editors. Elephants and savanna woodland ecosystems: a study from Chobe National Park, Botswana. Chichester, UK: Wiley-Blackwell and Zoological Society of London pp. 154–171.

[31] Augustine DJ, McNaughton SJ (1998) Ungulate effects on the functional species composition of plant communities: Herbivore selectivity and plant tolerance. J. Wildl. Manage. 62: 1165–1183.

[32] Van Auken OW (2000) Shrub invasions of North American semiarid grasslands. Annu. Rev. Ecol. Syst. 31: 197–215.

[33] Beck JL, Peek JM (2005) Diet composition, forage selection, and potential for forage competition among elk, deer, and livestock on aspen-sagebrush summer range. Rangeland Ecol. Manage. 58: 135–147.

[34] Cook JG (2002) Nutrition and food In: Toweill D. E. and Thomas J. W., editors. North American elk: Ecology and management. Washington, D.C.: Smithsonian Institution Press pp. 259–349.

[35] Baker DL, Hobbs NT (1982) Composition and quality of elk summer diets in Colorado. J. Wildl. Manage. 46: 694–703.

[36] Hobbs NT, Baker DL, Ellis JE, Swift DM (1981) Composition and Quality of Elk Winter Diets in Colorado. J. Wildl. Manage. 45: 156–171.

[37] Mysterud A (2000) Diet overlap among ruminants in Fennoscandia. Oecologia 124: 130–137. 28308406 10.1007/s004420050032

[38] Sankaran M, Augustine DJ, Ratnam J (2013) Native ungulates of diverse body sizes collectively regulate long-term woody plant demography and structure of a semi-arid savanna. J. Ecol. 101: 1389–1399.

[39] Staver AC, Bond WJ, February EC (2011) History matters: tree establishment variability and species turnover in an African savanna. Ecosphere 2: 49.

[40] McInnes PF, Naiman RJ, Pastor J, Cohen Y (1992) Effects of Moose Browsing on Vegetation and Litter of the Boreal Forest, Isle Royale, Michigan, USA. Ecology 73: 2059–2075.

[41] Reisner MD, Grace JB, Pyke DA, Doescher PS (2013) Conditions favouring *Bromus tectorum* dominance of endangered sagebrush steppe ecosystems. J. Appl. Ecol. 50: 1039–1049.

[42] Chambers JC, Roundy BA, Blank RR, Meyer SE, Whittaker A (2007) What makes Great Basin sagebrush ecosystems invasible by Bromus tectorum? Ecol. Monogr. 77: 117–145.

[43] Vavra M, Parks CG, Wisdom MJ (2007) Biodiversity, exotic plant species, and herbivory: The good, the bad, and the ungulate. For. Ecol. Manage. 246: 66–72.

[44] Kalisz S, Spigler RB, Horvitz CC (2014) In a long-term experimental demography study, excluding ungulates reversed invader’s explosive population growth rate and restored natives. Proc. Natl. Acad. Sci. USA 111: 4501–4506. 10.1073/pnas.1310121111 24616522 PMC3970537

[45] Manier DJ, Hobbs NT (2007) Large herbivores in sagebrush steppe ecosystems: livestock and wild ungulates influence structure and function. Oecologia 152: 739–750. 17375334 10.1007/s00442-007-0689-z

[46] Young TP, Okello BD, Kinyua D, Palmer TM (1998) KLEE: a long-term multi-species herbivore exclusion experiment in Laikipia, Kenya. Afr. J. Range Forage Sci. 14: 94–102.

[47] Porensky LM, Wittman SE, Riginos C, Young TP (2013) Herbivory and drought interact to enhance spatial patterning and diversity in a savanna understory. Oecologia 173: 591–602. 10.1007/s00442-013-2637-4 23494287

[48] Kay CE, Bartos DL (2000) Ungulate herbivory on Utah aspen: Assessment of long-term exclosures. J. Range Manage. 53: 145–153.

[49] Brookshire ENJ, Kauffman JB, Lytjen D, Otting N (2002) Cumulative effects of wild ungulate and livestock herbivory on riparian willows. Oecologia 132: 559–566. 28547642 10.1007/s00442-002-1007-4

[50] Pekin BK, Wisdom MJ, Endress BA, Naylor BJ, Parks CG (2014) Ungulate browsing maintains shrub diversity in the absence of episodic disturbance in seasonally-arid conifer forest. PLoS ONE 9: e86288 10.1371/journal.pone.0086288 24466006 PMC3900502

[51] Jones WB (1965) Response of major plant species to elk and cattle grazing in northwestern Wyoming. J. Range Manage. 18: 218–220.

[52] Bakker JD, Rudebusch F, Moore MM (2010) Effects of long-term livestock grazing and habitat on understory vegetation. West. N. Am. Nat. 70: 334–344.

[53] Davies KW, Svejcar TJ, Bates JD (2009) Interaction of historical and nonhistorical disturbances maintains native plant communities. Ecol. Appl. 19: 1536–1545. 19769101 10.1890/09-0111.1

[54] Camp MJ, Rachlow JL, Shipley LA, Johnson TR, Bockting KD (2014) Grazing in sagebrush rangelands in western North America: implications for habitat quality for a sagebrush specialist, the pygmy rabbit. Rangeland J. 36: 151–159.

[55] Frank DA, Groffman PM (1998) Ungulate vs. landscape control of soil C and N processes in grasslands of Yellowstone National Park. Ecology 79: 2229–2241.

[56] Augustine DJ, Frank DA (2001) Effects of migratory grazers on spatial heterogeneity of soil nitrogen properties in a grassland ecosystem. Ecology 82: 3149–3162.

[57] Kraaij T, Milton SJ (2006) Vegetation changes (1995–2004) in semi-arid Karoo shrubland, South Africa: Effects of rainfall, wild herbivores and change in land use. J. Arid Environ. 64: 174–192.

[58] Anderson TM, Ritchie ME, McNaughton SJ (2007) Rainfall and soils modify plant community response to grazing in Serengeti National Park. Ecology 88: 1191–1201. 17536405 10.1890/06-0399

[59] Rexroad EA, Beard KH, Kulmatiski A (2007) Vegetation responses to 35 and 55 years of native ungulate grazing in shrubsteppe communities. West. N. Am. Nat. 67: 16–25.

[60] Knick ST, Dobkin DS, Rotenberry JT, Schroeder MA, Vander Haegen WM, et al (2003) Teetering on the edge or too late? Conservation and research issues for avifauna of sagebrush habitats. Condor 105: 611–634.

[61] Noss RF, LaRoe III ET, Scott JM (1995) Endangered Ecosystems of the United States: A Preliminary Assessment of Loss and Degradation. National Biological Service Biological Report 28. Washington, D.C., USA: U.S. Department of the Interior.

[62] Connelly JW, Knick ST, Schroeder MA, Stiver SJ (2004) Conservation assessment of Greater Sage-grouse and sagebrush habitats. *Western Association of Fish and Wildlife Agencies. Unpublished report. Cheyenne, WY*.

[63] Miller RF, Knick ST, Pyke DA, Meinke CW, Hanser SE, et al (2011) Ch. 11: Characteristics of sagebrush habitats and limitations to long-term conservation Studies in Avian Biology, Ecology and conservation of greater sage-grouse: a landscape species and its habitats. University of California Press.

[64] NRCS (2013) The PLANTS Database (http://plants.usda.gov, 28 October 2013). National Plant Data Team, Greensboro, NC 27401–4901 USA. 10.1016/j.mgene.2013.11.003 25612187

[65] Herrick JE, van Zee JW, Havstad KM, Burkett LM, Whitford WG (2005) Monitoring Manual for Grassland, Shrubland and Savanna Ecosystems, Vol. II Las Cruces, New Mexico: Jornada Experimental Range.

[66] McCune B, Grace JB (2002) Analyis of ecological communities. Gleneden Beach, OR, USA: MjM Software Design.

[67] Pinheiro J, Bates D, DebRoy S, Sarkar D, the R Development Core Team (2013) nlme: Linear and Nonlinear Mixed Effects Models. R package version 3.

[68] Hedges LV (1981) Distributional theory for Glass’s estimator of effect size and related estimators. Journal of Educational Statistics 6.

[69] Cohen J (1988) Statistical Power Analysis for the Behavioral Sciences, 2nd edition Erlbaum.

[70] Nakagawa S, Cuthill IG (2007) Effect size, confidence interval and statistical significance: a practical guide for biologists. Biol. Rev. Camb. Philos. Soc. 82: 591–605. 17944619 10.1111/j.1469-185X.2007.00027.x

[71] Gurevitch J, Hedges LV (1999) Statistical issues in ecological meta-analyses. Ecology 80: 1142–1149.

[72] Porensky LM, Bucher SF, Veblen KE, Treydte AC, Young TP (2013) Megaherbivores and cattle alter edge effects around ecosystem hotspots in an African savanna. J. Arid Environ. 96: 55–63.

[73] Young TP, Palmer TM, Gadd ME (2005) Competition and compensation among cattle, zebras, and elephants in a semi-arid savanna in Laikipia, Kenya. Biol. Conserv. 122: 351–359.

[74] Pajunen A, Virtanen R, Roininen H (2012) Browsing-mediated shrub canopy changes drive composition and species richness in forest-tundra ecosystems. Oikos 121: 1544–1552.

[75] Riginos C (2009) Grass competition suppresses savanna tree growth across multiple demographic stages. Ecology 90: 335–340. 19323216 10.1890/08-0462.1

[76] Staver AC, Bond WJ, Stock WD, van Rensburg SJ, Waldram MS (2009) Browsing and fire interact to suppress tree density in an African savanna. Ecol. Appl. 19: 1909–1919. 19831079 10.1890/08-1907.1

[77] Scholes RJ, Archer SR (1997) Tree-grass interactions in savannas. Annu. Rev. Ecol. Syst. 28: 517–544.

[78] Eldridge DJ, Bowker MA, Maestre FT, Roger E, Reynolds JF, et al (2011) Impacts of shrub encroachment on ecosystem structure and functioning: towards a global synthesis. Ecol. Lett. 14: 709–722. 10.1111/j.1461-0248.2011.01630.x 21592276 PMC3563963

[79] Crawford JA, Olson RA, West NE, Mosley JC, Schroeder MA, et al (2004) Ecology and management of sage-grouse and sage-grouse habitat. J. Range Manage. 57: 2–19.

[80] Boyd CS, Svejcar TJ (2011) The influence of plant removal on succession in Wyoming big sagebrush. J. Arid Environ. 75: 734–741.

[81] Chambers JC, Bradley BA, Brown CS, D’Antonio C, Germino MJ, et al (2014) Resilience to Stress and Disturbance, and Resistance to *Bromus tectorum* L. Invasion in Cold Desert Shrublands of Western North America. Ecosystems 17: 360–375.

[82] Poulos JM, Rayburn AP, Schupp EW (2014) Simultaneous, independent, and additive effects of shrub facilitation and understory competition on the survival of a native forb (Penstemon palmeri). Plant Ecol. 215: 417–426.

[83] Prevey JS, Germino MJ, Huntly NJ (2010) Loss of foundation species increases population growth of exotic forbs in sagebrush steppe. Ecol. Appl. 20: 1890–1902. 21049877 10.1890/09-0750.1

[84] Leger EA, Goergen EM, de Queiroz TF (2014) Can native annual forbs reduce Bromus tectorum biomass and indirectly facilitate establishment of a native perennial grass? J. Arid Environ. 102: 9–16.

[85] Yeo JJ (2005) Effects of grazing exclusion on rangeland vegetation and soils, East Central Idaho. West. N. Am. Nat. 65: 91–102.

[86] Schlaepfer DR, Lauenroth WK, Bradford JB (2014) Natural regeneration processes in big sagebrush (Artemisia tridentata). Rangeland Ecol. Manage. 67: 344–357.

[87] Petersen CA, Villalba JJ, Provenza FD (2014) Influence of Experience on Browsing Sagebrush by Cattle and Its Impacts on Plant Community Structure. Rangeland Ecol. Manage. 67: 78–87.

[88] Bryant JP, Provenza FD, Pastor J, Reichardt PB, Clausen TP, et al (1991) Interactions between woody plants and browsing mammals mediated by secondary metabolites. Annu. Rev. Ecol. Syst. 22: 431–446.

[89] Frank DA, McNaughton SJ (1993) Evidence for the Promotion of Aboveground Grassland Production by Native Large Herbivores in Yellowstone National Park. Oecologia 96: 157–161. 28313410 10.1007/BF00317727

[90] Bagchi S, Ritchie M (2010) Herbivore effects on above- and belowground plant production and soil nitrogen availability in the Trans-Himalayan shrub-steppes. Oecologia 164: 1075–1082. 10.1007/s00442-010-1690-5 20585808

[91] Chambers JC, Wisdom MJ (2009) Priority research and management issues for the imperiled Great Basin of the western United States. Restor. Ecol. 17: 707–714.

[92] Hobbs RJ (2001) Synergisms among habitat fragmentation, livestock grazing, and biotic invasions in southwestern Australia. Conserv. Biol. 15: 1522–1528.

[93] Smith MD, Knapp AK (1999) Exotic plant species in a C-4-dominated grassland: invasibility, disturbance, and community structure. Oecologia 120: 605–612. 28308312 10.1007/s004420050896

[94] Polley HW, Briske DD, Morgan JA, Wolter K, Bailey DW, et al (2013) Climate Change and North American Rangelands: Trends, Projections, and Implications. Rangeland Ecol. Manage. 66: 493–511.

[95] Tilman D (1997) Community invasibility, recruitment limitation, and grassland biodiversity. Ecology 78: 81–92.

[96] Okin GS, Parsons AJ, Wainwright J, Herrick JE, Bestelmeyer BT, et al (2009) Do changes in connectivity explain desertification? Bioscience 59: 237–244.

[97] Bowker MA (2007) Biological soil crust rehabilitation in theory and practice: An underexploited opportunity. Restor. Ecol. 15: 13–23.

[98] Read CF, Duncan DH, Vesk PA, Elith J (2014) Biocrust morphogroups provide an effective and rapid assessment tool for drylands. J. Appl. Ecol. Early View. 10.1111/1365-2664.12336 PMC4286204 25598550

[99] Richardson DM, Pysek P (2006) Plant invasions: merging the concepts of species invasiveness and community invasibility. Prog. Phys. Geogr. 30: 409–431.

[100] Allen PS, Meyer SE (2014) Community structure affects annual grass weed invasion during restoration of a shrub-steppe ecosystem. Inv. Plant Sci. Manage. 7: 1–13.

[101] Ponzetti JM, McCune B, Pyke DA (2007) Biotic soil crusts in relation to topography, cheatgrass and fire in the Columbia Basin, Washington. Bryologist 110: 706–722.

[102] Leger EA (2008) The adaptive value of remnant native plants in invaded communities: An example from the Great Basin. Ecol. Appl. 18: 1226–1235. 18686583 10.1890/07-1598.1

[103] Humphrey LD, Schupp EW (2004) Competition as a barrier to establishment of a native perennial grass (Elymus elymoides) in alien annual grass (Bromus tectorum) communities. J. Arid Environ. 58: 405–422.

[104] Milchunas DG, Lauenroth WK, Chapman PL, Kazempour MK (1989) Effects of grazing, topography, and precipitation on the structure of a semiarid grassland. Vegetatio 80: 11–23.

