# Supplementary material for: Contrasting Effects of Different Mammalian Herbivores on Sagebrush Plant Communities
Source: PLoS One. 2015 Feb 11;10(2):e0118016. doi: 10.1371/journal.pone.0118016 (PMC4324772; doi:10.1371/journal.pone.0118016)
Supplement: S2 File — Plant frequencies for five transects (n = five 1 m x 1 m quadrats per transect) at each of three treatments (DX = no large ungulates allowed, LX = cattle excluded, accessible by wild ungulates only, OX = accessible by cattle and wild ungulates) for each of three sites (Kate, Dip, Neponset). Species codes follow USDA Plant Database codes (http://plants.usda.gov/); AF53 is an unidentified annual forb. (DOCX) [file pone.0118016.s002.docx]

| Site | Trt | Transect | ACHY | AF53 | AGCR | ARTR2 | BRTE | CHVI8 | COWR | ELEL5 | HECOC8 | LIDA | OPPO | ORLU2 | PASM | PHHO | PHLO2 | POSE | PSSP6 | TECA | TOIN |
| --- | --- | --- | --- | --- | --- | --- | --- | --- | --- | --- | --- | --- | --- | --- | --- | --- | --- | --- | --- | --- | --- |
| Kate | DX | 1 | 1 | 0 | 1 | 4 | 0 | 5 | 0 | 1 | 1 | 3 | 1 | 1 | 1 | 5 | 1 | 5 | 0 | 1 | 1 |
| Kate | DX | 2 | 2 | 0 | 2 | 4 | 0 | 3 | 0 | 1 | 3 | 4 | 1 | 2 | 0 | 3 | 1 | 4 | 2 | 0 | 0 |
| Kate | DX | 3 | 2 | 0 | 1 | 4 | 0 | 2 | 0 | 2 | 3 | 3 | 0 | 1 | 0 | 5 | 1 | 5 | 1 | 0 | 1 |
| Kate | DX | 4 | 4 | 0 | 1 | 5 | 0 | 5 | 0 | 4 | 1 | 2 | 0 | 1 | 1 | 5 | 0 | 4 | 0 | 1 | 0 |
| Kate | DX | 5 | 2 | 0 | 2 | 5 | 0 | 4 | 0 | 4 | 3 | 3 | 1 | 0 | 0 | 4 | 1 | 5 | 2 | 0 | 0 |
| Kate | LX | 1 | 0 | 0 | 0 | 5 | 1 | 4 | 0 | 2 | 0 | 0 | 0 | 0 | 0 | 4 | 2 | 5 | 1 | 0 | 0 |
| Kate | LX | 2 | 1 | 0 | 1 | 5 | 0 | 4 | 0 | 4 | 1 | 0 | 1 | 0 | 1 | 2 | 2 | 5 | 0 | 1 | 1 |
| Kate | LX | 3 | 1 | 1 | 0 | 5 | 1 | 2 | 0 | 3 | 2 | 0 | 0 | 0 | 0 | 3 | 2 | 5 | 2 | 0 | 1 |
| Kate | LX | 4 | 0 | 0 | 0 | 5 | 0 | 5 | 0 | 3 | 1 | 0 | 0 | 0 | 1 | 4 | 3 | 5 | 0 | 0 | 0 |
| Kate | LX | 5 | 0 | 0 | 1 | 4 | 0 | 5 | 0 | 2 | 1 | 0 | 0 | 0 | 0 | 3 | 2 | 5 | 0 | 0 | 1 |
| Kate | OX | 1 | 1 | 0 | 1 | 5 | 0 | 4 | 1 | 2 | 0 | 0 | 1 | 1 | 2 | 3 | 0 | 5 | 1 | 0 | 0 |
| Kate | OX | 2 | 0 | 0 | 1 | 5 | 0 | 4 | 1 | 3 | 1 | 0 | 0 | 0 | 2 | 4 | 4 | 5 | 2 | 0 | 0 |
| Kate | OX | 3 | 0 | 0 | 2 | 5 | 0 | 4 | 0 | 2 | 1 | 0 | 0 | 3 | 1 | 3 | 4 | 4 | 1 | 1 | 1 |
| Kate | OX | 4 | 0 | 0 | 0 | 5 | 0 | 5 | 0 | 1 | 0 | 0 | 1 | 2 | 0 | 5 | 0 | 5 | 0 | 0 | 3 |
| Kate | OX | 5 | 0 | 0 | 2 | 3 | 0 | 5 | 0 | 4 | 1 | 1 | 0 | 1 | 2 | 2 | 2 | 5 | 1 | 0 | 0 |
| Nep | DX | 1 | 2 | 0 | 0 | 5 | 0 | 4 | 0 | 2 | 3 | 1 | 0 | 2 | 0 | 4 | 3 | 5 | 2 | 1 | 0 |
| Nep | DX | 2 | 1 | 0 | 0 | 4 | 0 | 3 | 0 | 2 | 3 | 0 | 0 | 0 | 0 | 0 | 3 | 4 | 1 | 0 | 0 |
| Nep | DX | 3 | 3 | 0 | 0 | 4 | 0 | 3 | 0 | 1 | 2 | 1 | 0 | 2 | 0 | 4 | 3 | 5 | 1 | 0 | 1 |
| Nep | DX | 4 | 1 | 1 | 0 | 5 | 0 | 3 | 2 | 1 | 2 | 0 | 1 | 1 | 0 | 3 | 5 | 4 | 0 | 0 | 0 |
| Nep | DX | 5 | 1 | 0 | 1 | 5 | 0 | 4 | 0 | 1 | 0 | 0 | 1 | 0 | 0 | 2 | 2 | 4 | 2 | 0 | 0 |
| Nep | LX | 1 | 2 | 0 | 0 | 5 | 0 | 4 | 0 | 3 | 1 | 0 | 1 | 1 | 1 | 3 | 1 | 5 | 2 | 0 | 1 |
| Nep | LX | 2 | 2 | 0 | 1 | 5 | 0 | 5 | 0 | 1 | 1 | 0 | 0 | 0 | 1 | 2 | 2 | 5 | 0 | 0 | 0 |
| Nep | LX | 3 | 1 | 0 | 1 | 5 | 0 | 4 | 0 | 3 | 2 | 0 | 0 | 2 | 0 | 1 | 3 | 4 | 1 | 0 | 0 |
| Nep | LX | 4 | 3 | 0 | 1 | 5 | 0 | 4 | 1 | 3 | 2 | 0 | 1 | 0 | 0 | 2 | 5 | 3 | 2 | 0 | 0 |
| Nep | LX | 5 | 3 | 1 | 2 | 4 | 0 | 5 | 0 | 2 | 3 | 0 | 1 | 1 | 0 | 3 | 2 | 5 | 1 | 1 | 1 |
| Nep | OX | 1 | 1 | 0 | 1 | 4 | 0 | 5 | 0 | 2 | 5 | 0 | 0 | 1 | 0 | 5 | 0 | 5 | 0 | 0 | 2 |
| Nep | OX | 2 | 3 | 0 | 0 | 5 | 0 | 2 | 3 | 1 | 3 | 0 | 0 | 4 | 0 | 2 | 3 | 5 | 0 | 0 | 0 |
| Nep | OX | 3 | 2 | 1 | 0 | 5 | 0 | 5 | 4 | 1 | 3 | 0 | 0 | 2 | 0 | 4 | 1 | 5 | 0 | 2 | 0 |
| Nep | OX | 4 | 3 | 1 | 0 | 4 | 0 | 3 | 1 | 1 | 2 | 0 | 0 | 3 | 0 | 2 | 3 | 5 | 1 | 1 | 0 |
| Nep | OX | 5 | 4 | 0 | 1 | 5 | 0 | 4 | 2 | 3 | 3 | 0 | 1 | 3 | 0 | 1 | 3 | 5 | 0 | 0 | 0 |
| Dip | DX | 1 | 2 | 0 | 1 | 5 | 1 | 4 | 2 | 4 | 1 | 0 | 0 | 0 | 0 | 2 | 0 | 5 | 0 | 3 | 2 |
| Dip | DX | 2 | 2 | 0 | 1 | 5 | 0 | 5 | 1 | 1 | 0 | 0 | 0 | 1 | 0 | 3 | 0 | 5 | 0 | 0 | 0 |
| Dip | DX | 3 | 0 | 0 | 2 | 5 | 0 | 5 | 2 | 3 | 0 | 0 | 1 | 1 | 0 | 3 | 0 | 4 | 0 | 2 | 2 |
| Dip | DX | 4 | 2 | 0 | 0 | 5 | 0 | 5 | 1 | 3 | 0 | 0 | 0 | 1 | 0 | 1 | 0 | 4 | 1 | 4 | 0 |
| Dip | DX | 5 | 0 | 1 | 0 | 4 | 1 | 4 | 0 | 4 | 0 | 0 | 0 | 2 | 1 | 3 | 3 | 4 | 1 | 2 | 1 |
| Dip | LX | 1 | 1 | 1 | 1 | 5 | 0 | 4 | 1 | 2 | 3 | 0 | 1 | 0 | 1 | 1 | 0 | 4 | 1 | 2 | 2 |
| Dip | LX | 2 | 2 | 1 | 0 | 5 | 2 | 1 | 1 | 5 | 3 | 0 | 0 | 0 | 0 | 0 | 0 | 3 | 1 | 2 | 1 |
| Dip | LX | 3 | 1 | 1 | 2 | 4 | 3 | 5 | 2 | 1 | 4 | 0 | 1 | 0 | 2 | 0 | 0 | 3 | 0 | 2 | 0 |
| Dip | LX | 4 | 1 | 1 | 0 | 5 | 3 | 4 | 1 | 2 | 3 | 0 | 1 | 0 | 0 | 1 | 0 | 5 | 0 | 2 | 0 |
| Dip | LX | 5 | 2 | 1 | 0 | 5 | 2 | 3 | 0 | 1 | 2 | 0 | 0 | 0 | 0 | 1 | 1 | 5 | 0 | 1 | 0 |
| Dip | OX | 1 | 2 | 1 | 0 | 4 | 0 | 4 | 1 | 2 | 2 | 0 | 0 | 2 | 0 | 2 | 2 | 4 | 1 | 1 | 1 |
| Dip | OX | 2 | 1 | 0 | 0 | 5 | 0 | 4 | 2 | 1 | 3 | 0 | 0 | 1 | 0 | 3 | 1 | 4 | 0 | 4 | 1 |
| Dip | OX | 3 | 3 | 1 | 1 | 2 | 1 | 5 | 1 | 0 | 3 | 0 | 0 | 0 | 0 | 1 | 0 | 5 | 0 | 4 | 1 |
| Dip | OX | 4 | 1 | 0 | 1 | 4 | 1 | 5 | 1 | 1 | 1 | 0 | 0 | 1 | 1 | 0 | 1 | 5 | 0 | 3 | 1 |
| Dip | OX | 5 | 1 | 0 | 0 | 3 | 2 | 3 | 0 | 1 | 3 | 0 | 2 | 0 | 0 | 1 | 1 | 5 | 1 | 6 | 0 |
